# Transcriptomic and biochemical investigations support the role of rootstock-scion interaction in grapevine berry quality

**DOI:** 10.1186/s12864-020-06795-5

**Published:** 2020-07-08

**Authors:** A. Zombardo, C. Crosatti, P. Bagnaresi, L. Bassolino, N. Reshef, S. Puccioni, P. Faccioli, A. Tafuri, M. Delledonne, A. Fait, P. Storchi, L. Cattivelli, E. Mica

**Affiliations:** 1 CREA Research Centre for Viticulture and Enology, viale Santa Margherita 80, 52100 Arezzo, Italy; 2 Department of Agriculture, Food, Environment and Forestry (DAGRI), University of Florence, Piazzale delle Cascine 18, 50144 Florence, Italy; 3 CREA Research Centre for Genomics and Bioinformatics, via San Protaso 302, 29017 Fiorenzuola d’Arda, PC Italy; 4 CREA Research Centre for Cereal and Industrial Crops, via di Corticella 133, 40128 Bologna, Italy; 5 French Associates institute for Agriculture and Biotechnology of Drylands, The Jacob Blaustein Institutes for Desert Research, Ben-Gurion University of the Negev, Midreshet Ben Gurion, 84990 Israel; 6 Present address: Department of Food Science, Cornell University, Ithaca, NY 14853 USA; 7 Department of Biotechnologies, University of Verona, Strada le Grazie 15, 37134 Verona, Italy

**Keywords:** Grapevine, *Vitis vinifera*, Rootstock, RNA-seq, miRNA, Transcriptomic, Berry ripening, Secondary metabolism

## Abstract

**Background:**

In viticulture, rootstock genotype plays a critical role to improve scion physiology, berry quality and to adapt grapevine (*Vitis vinifera* L.) to different environmental conditions. This study aimed at investigating the effect of two different rootstocks (1103 Paulsen - P - and Mgt 101–14 - M) in comparison with not grafted plants - NGC - on transcriptome (RNA-seq and small RNA-seq) and chemical composition of berry skin in *Pinot noir*, and exploring the influence of rootstock-scion interaction on grape quality. Berry samples, collected at veraison and maturity, were investigated at transcriptional and biochemical levels to depict the impact of rootstock on berry maturation.

**Results:**

RNA- and miRNA-seq analyses highlighted that, at veraison, the transcriptomes of the berry skin are extremely similar, while variations associated with the different rootstocks become evident at maturity, suggesting a greater diversification at transcriptional level towards the end of the ripening process. In the experimental design, resembling standard agronomic growth conditions, the vines grafted on the two different rootstocks do not show a high degree of diversity. In general, the few genes differentially expressed at veraison were linked to photosynthesis, putatively because of a ripening delay in not grafted vines, while at maturity the differentially expressed genes were mainly involved in the synthesis and transport of phenylpropanoids (e.g. flavonoids), cell wall loosening, and stress response. These results were supported by some differences in berry phenolic composition detected between grafted and not grafted plants, in particular in resveratrol derivatives accumulation.

**Conclusions:**

Transcriptomic and biochemical data demonstrate a stronger impact of 1103 Paulsen rootstock than Mgt 101–14 or not grafted plants on ripening processes related to the secondary metabolite accumulations in berry skin tissue. Interestingly, the *MYB14* gene, involved in the feedback regulation of resveratrol biosynthesis was up-regulated in 1103 Paulsen thus supporting a putative greater accumulation of stilbenes in mature berries.

## Background

Grapevine (*Vitis vinifera*) is one of the oldest and most economically important fruit crops and is well adapted to grow in a wide range of climatic conditions. It is a perennial plant mainly cultivated for wine production or food (fresh fruit, juice or raisins). In recent years, following the complete sequencing of its genome [1, 2], it has become a model plant for non-climacteric fruit research.

In *Vitis vinifera* cultivation, it is almost mandatory to graft the vines on rootstock derived from American *Vitis* species resistant to phylloxera (*Daktulosphaira vitifoliae* Fitch*,* a soil-dwelling aphid), a pest that spread in Europe at the end of the nineteenth century and devastated a large portion of cultivated vineyards. Since its introduction, grafting represents the only form of biological control available against this plague [3]. During the selection of the different rootstock genotypes, several additional traits have been fixed by breeders to provide to the scion higher tolerance to environmental adversities and abiotic stresses, such as soil limestone, high salinity, stagnation, drought, and frost [4, 5].

The rootstock acts as an interface between the scion and the soil ecosystem [6] and its role on the scion’s physiology is a highly debated subject in the literature. According to some authors, the rootstock modifies source-sink relations, influencing vine’s performances [7–9], whereas other studies suggest that the rootstock has a minor effect on the physiological behavior of the scion, whose genotype is the main factor that concretely determines the shoot vegetative development and the characteristics of the grapes produced [10, 11].

The molecular processes governing rootstock-scion interaction remain largely unknown and deepening this topic is rather difficult because the grafting implies huge structural changes and hydraulic integration [12] through the reprogramming of gene expression and protein translation. Moreover, grafting is perceived as a considerable trauma by the plant that triggers some defense and stress response mechanisms [13], such as the expression of genes involved in cell wall synthesis, hormone signaling and secondary metabolism [14]. According to recent discoveries, besides small molecules (such as water, ions, amino acids, and hormones), also some macromolecules (such as mRNAs, proteins, but most of all miRNAs) are mobile through the plant across the graft union [15–19]. It is currently known that the rootstock can alter the gene expression in the scion, especially in the presence of stress, disease or limiting factors. Several transcriptome changes are related to the phenylpropanoid pathway genes, like those responsible for stilbene and flavonoid biosynthesis [13, 20–25].

Stilbenes and flavonoids are secondary metabolites, both derived from the same precursor, the amino acid phenylalanine. These two classes of phenolic compounds synthesized through the phenylpropanoid pathway share some initial steps [26]. Stilbenes are naturally present in grapes [27], and their synthesis increases in case of pathogen attack or at the onset of abiotic stresses. The main stilbene in grapes and wines is resveratrol, a molecule that is gaining attention for its nutraceutical and pharmacologic properties [28, 29]. Flavonoids are the most effective antioxidants in grapes and are located mainly in berry skins and as tannins in seeds, in considerable concentrations [26, 30]. The flavonoid composition of grapes (anthocyanins, flavonols, and simple flavanols or proanthocyanidins) is essential for wine quality, given their great influence on the organoleptic characteristics and the aging aptitude. The accumulation of phenolic compounds in grapes can vary widely, depending on environmental conditions, nutrient availability, water status, canopy thickness and cluster exposure [31–33] and, according to some authors, there is also a possible influence of the rootstock genotype [14, 22, 23, 34].

The transcriptional or post-transcriptional regulation of the structural genes involved in the phenylpropanoid biosynthetic pathway is controlled in plants at different levels by several mechanisms, such as transcription factors, for example MYBs [35] or RNA interference, where miRNAs are key players [36, 37]. In grapevine, R2R3-MYBs are by far the most important class of MYB that controls flavonoid and stilbene accumulations during ripening, at the different spatial-temporal level [30].

miRNAs are small non-coding RNAs (19–24 nt long), coded by specific MIR genes, that perform Post-Transcriptional Gene Silencing (PTGS), through a sequence-specific down-regulation of gene expression [38–40]. In recent years, some studies have revealed the central role of miRNAs in grapevine metabolism and development [38, 40–44]. Grafting can alter miRNAs abundance in the scion, as their movement through the vascular system is coupled with stress signals, causing changes in the final phenotype [15, 45].

This research aimed at investigating how different rootstocks influence gene expression and phenotype in berry skin, where secondary metabolites accumulate, to find out their actual effects on the quality of the grapes produced. The project was set up in an experimental system of potted *Pinot noir* grapevines, that included plants grafted on two rootstocks with opposite characteristics (1103 Paulsen, highly vigorous and tolerant to drought, and Mgt 101–14, less vigorous and susceptible to drought), as well as not grafted plants, to test the rootstock effect in vines grown with identical agronomic conditions and water supply. Gene expression, both mRNA and small RNA, was evaluated on berry skins at two specific time points (veraison and maturity), and data were analyzed searching for the expression profile of some miRNAs and target transcripts correlated to the secondary metabolism. Alongside the genetic analysis, chemical analyses on grape skins were performed to assess the accumulation and composition of phenolic compounds, at the onset of ripening (veraison) and maturity.

## Results

### Weather conditions

The data recorded during the year 2012 (from April 1st – DOY 92, to October 31st – DOY 305) in the experimental area are reported in Additional file 1. In general, the growing season was warm, with 1450 GDDs accumulated in the period April 1st (DOY 92) – August 22nd (DOY 235, harvest date) and a total amount of rainfall of 217 mm. Considering the interval between veraison (T1 – DOY 214) and maturity (T2 – DOY 235) samplings only, the temperatures were quite high, with the following values recorded: Average T_max_ = 35.6 °C; Average T_avg_ = 26.6 °C; Average T_min_ = 16.2 °C. Compared to the historical data (1951–2011) of the climate region of Arezzo (www.sir.toscana.it), the daily minimum temperatures recorded in the same reference period were consistent (Average T_min_ = 16.1 °C), while both the daily average temperatures and the daily maximum temperatures were few degrees higher (Average T_avg_ = 24.2 °C; Average T_max_ = 32.3 °C). In fact, a good part of the total GDDs (347) was accumulated between T1 and T2. During this time frame (21 days), only a few rain events were recorded, with 7.8 mm rainfall, much lower than the historical average (1951–2011) of 44 mm (found at: www.sir.toscana.it).

### RNA-seq and reads mapping to grapevine genome

Eighteen RNAseq libraries were sequenced producing on average 21 million reads (Additional file 2). Quality filtered reads were mapped to the *Vitis vinifera* 12x.25 reference genome. Pearson correlation coefficients within biological replicates were always above 0,97 (Additional file 3), indicating a high level of reproducibility.

**Fig. 1:**
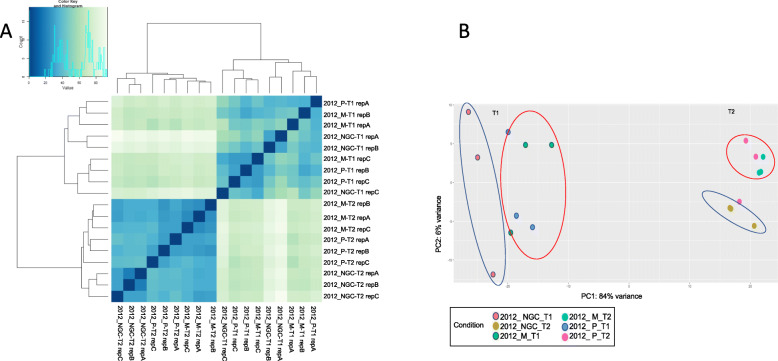
Panel **a**: Hierarchical cluster analysis (HCA) of all samples sequenced by RNA-seq. Heatmaps reporting clustering of all samples were generated upon rlog-transformation of DESeq2-normalized expression data. Color key scheme: X axis reports euclidean distances among samples, Y axis reports the number of times a color/value is represented in the graph. Panel **b**: Principal Component Analysis (PCA) of the samples sequenced by RNA-seq. X-axis represents first component, Y-axis the second component. Dots with the same color indicate same sample, different replicates. Blue ovals enclose NGC samples, red ovals enclose grafted samples. Sample names: M = Mgt 101–14; *P* = 1103 Paulsen; NGC = not grafted control; Replicate A, B, C. T1 = veraison; T2 = maturity. (PDF 264 kb)

Hierarchical Clustering analysis with rlog transformed data was used to evaluate sample correlation. Fig. 1 A clearly shows that the berry developmental stage was the strongest driving force: samples at T1 (veraison) were separated from samples at T2 (maturity). Moreover, at T2, not grafted plants (NGC) were grouped together apart from the grafted ones. As expected, PCA (Fig. 1 b) revealed again a clear distinction between samples at T1 and samples at T2 as well as a separation between NGC and grafted samples, both at veraison and, above all, at maturity.

### Differential expression analyses

**Fig. 2:**
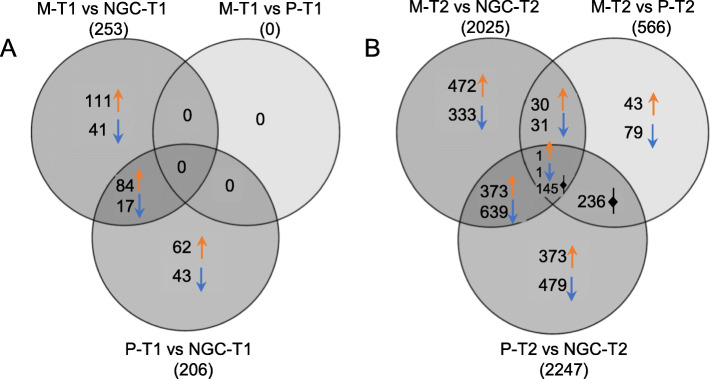
Venn diagrams of genes differentially expressed between the three root systems, at the same developmental stage (Panel **a**: T1 = veraison, Panel **b**: T2 = maturity). Total numbers of DEGs are in brackets, number of up- and down-regulated genes are indicated per each sub-set besides colored arrows. DEGs were called setting the FDR threshold at 0.05. Sample names: M = Mgt 101–14; *P* = 1103 Paulsen; NGC = not grafted control (PDF 48 kb)

Pairwise comparison between the grafted vines (M and P) and the not grafted (NGC), at the same developmental stage, were performed to evaluate the rootstock effects on berry skin transcriptome. The number of DEGs in the six comparisons, M-T1 vs NGC-T1; P-T1 vs NGC-T1; M-T1 vs P-T1; M-T2 vs NGC-T2; P-T2 vs NGC-T2; M-T2 vs P-T2, was highly variable ranging from zero to 2247 (Fig. 2 and Additional file 4). In general, we can describe two major trends. First, comparing berry skins from vines with different rootstock/scion combinations we obtained much fewer DEGs at T1 than at T2, indicating stronger differences in the transcriptome towards the end of the ripening process. Second, M and P grafted plants were more similar to each other than to NGC plants, suggesting that the grafting per se had a significant impact on the transcriptome profile, and that non-stressful conditions did not create such environmental cues able to bring out remarkable differences among the two different rootstocks.

Among DEGs at T1, most genes were up-regulated in NGC when compared to M or P plants (77 and 71% respectively). At T2, the percentages were almost the opposite: 57 and 63% of DEGs were down-regulated in NGC compared to M or P plants, respectively. Comparing P with M, genes were mostly (66%) up-regulated in 1103 Paulsen. In general, the log_2_ fold change was ranging between − 4.8 and + 3.2.

**Fig. 3:**
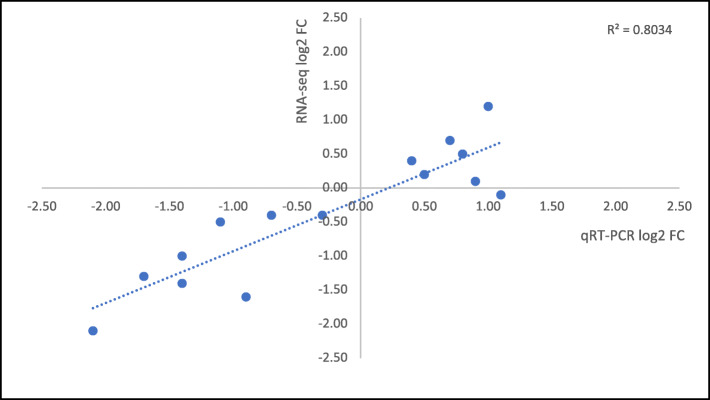
Scatter Plot showing correlation between log2 Fold Change obtained via RNAseq (Y axis) and qRT-PCR (X axis) data. Regression line is plotted, and R^2^ is shown (PDF 35 kb)

**Fig. 4:**
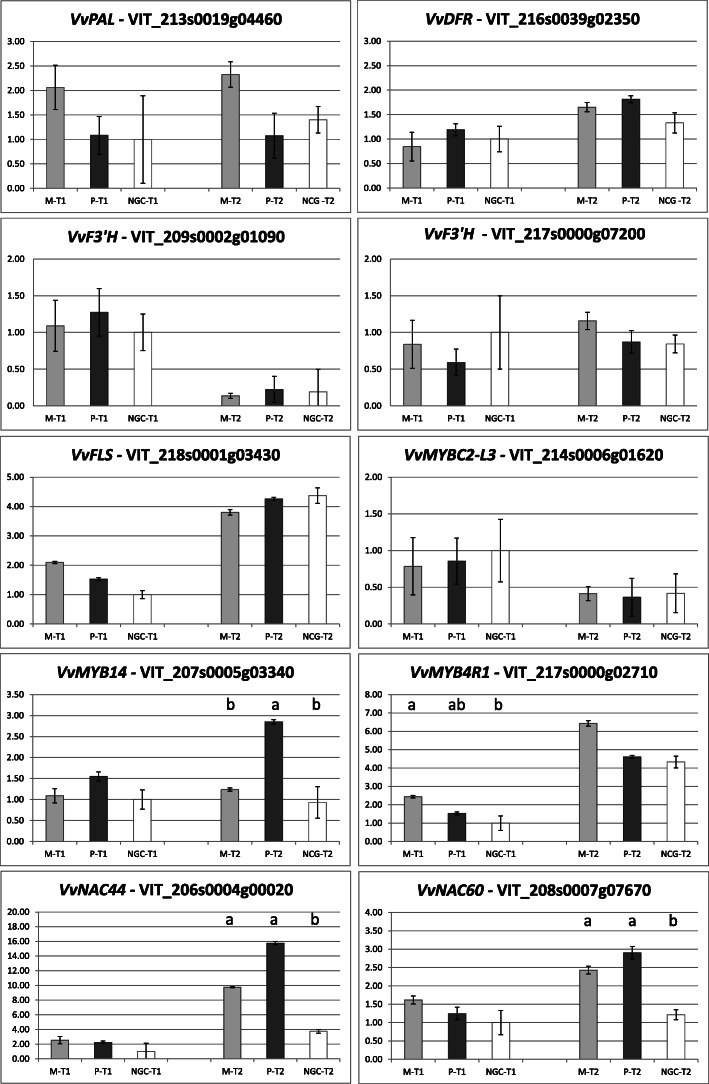
Expression profiles of the 10 selected genes coding for structural genes and transcription factors obtained by qRT-PCR, calculation from Ct value with the 2^-ΔΔCt^ method (the bars indicate the standard error, different letters indicate statistically different samples (one-way ANOVA, *P* value < 0.05, mean values separated by LSD multiple range test, 95% confidence interval) at each ripening time). Sample names: M = Mgt 101–14; *P* = 1103 Paulsen; NGC = not grafted control; T1 = veraison; T2 = maturity (PDF 446 kb)

To validate the RNA-seq data, we selected 10 genes to be analyzed by qRT-PCR. All the genes chosen are specifically involved in key points of the phenylpropanoid pathway, as structural genes (*PAL - PHENYLALANINE AMMONIA LYASE*, 2 copies of *F3’H - FLAVONOID 3′-HYDROXYLASE*, *FLS - FLAVONOL SYNTHASE*, and *DFR - DIHYDROFLAVONOL-4-REDUCTASE*) or transcription factors belonging to MYB (*MYB14, MYB4R1,* and *MYBC2-L3*) and *NAC* (*NAC44*, and *NAC60*) gene families. qRT-PCR reactions results were compared with the DESeq2 pairwise comparison outputs. The fold change values obtained by qRT-PCR confirmed those obtained by RNA-seq, validating the results and the technique (Fig. 3, and Fig. 4).

### Gene ontology enrichment

**Fig. 5:**
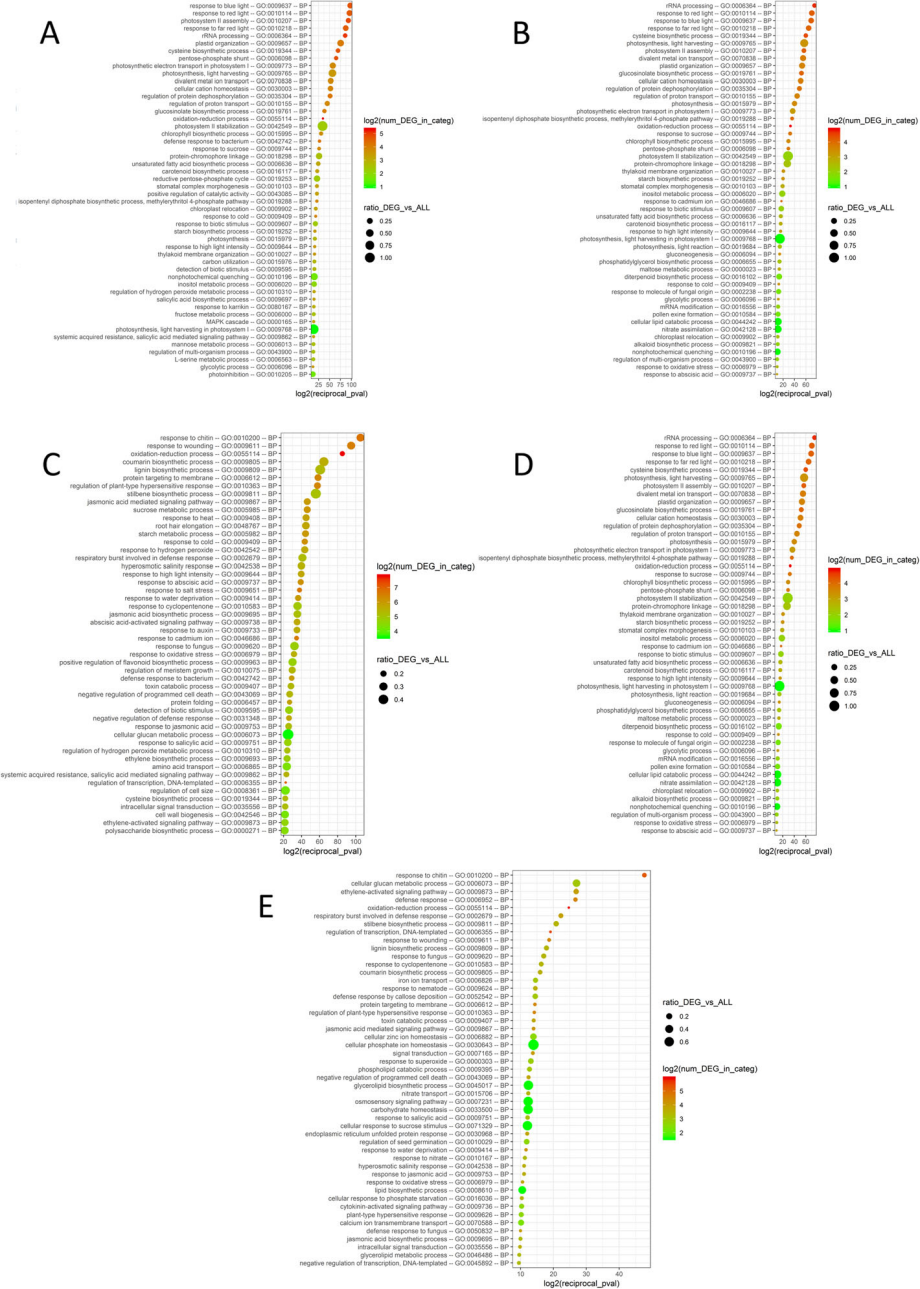
GO enrichment for Biological Process (BP) domain in the comparison of the transcriptomes of grafted (M - Mgt 101–14 or P - 1103 Paulsen) and not grafted control (NGC) plants, at veraison (T1) or maturity (T2). Top 50 GO, ranked based on *p*-value, are shown. Panel **a**: GO enriched in the comparison M -T1 vs NGC – T1; Panel **b**: GO enriched in the comparison P-T1 vs NGC-T1; Panel **c**: GO enriched in the comparison M-T2 vs NGC-T2; Panel **d**: GO enriched in the comparison P-T2 vs NGC-T2; Panel **e**: GO enriched in the comparison M-T2 vs P-T2. GO IDs and corresponding GO terms are as specified in the Y-axis. GOs are sorted according to decreasing log_2_ (1/*p*-value) on the X-axis. The absolute number of DEGs that matched the GO term (log_2_-transformed) is indicated by the color of each spot, whereas the size of each spot shows the ratio of DEGs versus all grapevine genes matching the same considered GO term (PDF 5350 kb)

To gain insights into the main metabolic and signaling pathways involved in the considered comparisons, we conducted GO enrichment analysis. Biological process enrichment analyses revealed that, at T1, there were 58 GO terms significantly over-represented in M vs NGC and 56 GO terms in the comparison P vs NGC (Additional file 5, and Fig. 5). Of these, 42 were shared between the comparisons and were mainly related to photosynthetic components and biotic/abiotic stress response.

More interestingly, at T2, the number of GO terms enriched in the performed comparisons were more abundant. We retrieved 203 and 168 GO terms (biological processes) when comparing M and P with NGC, respectively, and 49 GO terms comparing the M vs P plants. Thirty-four GO terms were shared among the three comparisons.

It is worth noting that 68 GO are specific to the M-T2 vs NGC-T2 comparison, and among them, we recovered four biological processes referred to fruit ripening (GO:0009835, GO:0045490), and its consecutive cell wall modification processes (GO:0071555, GO:0042545, GO:0046274, GO:0009831), plus two related to cinnamic acid (GO:0009800) and alkaloid (GO:0009821) biosynthesis. Interestingly, there are also GO terms related to drought stress response (GO:0009269, GO:0009819, GO:0006833), a biological process that has a key role during grape maturation, considering that it occurs during a season characterized by high daily temperatures, low rainfall rates, and more frequent drought events. For P-T2 vs NGC-T2, we retrieved two GO, uniquely enriched in this comparison, related to pigment and anthocyanin accumulation (GO:0046148, GO:0031537), peculiar processes that play a key role in winemaking and in the aging attitude of the wines.

**Fig. 6:**
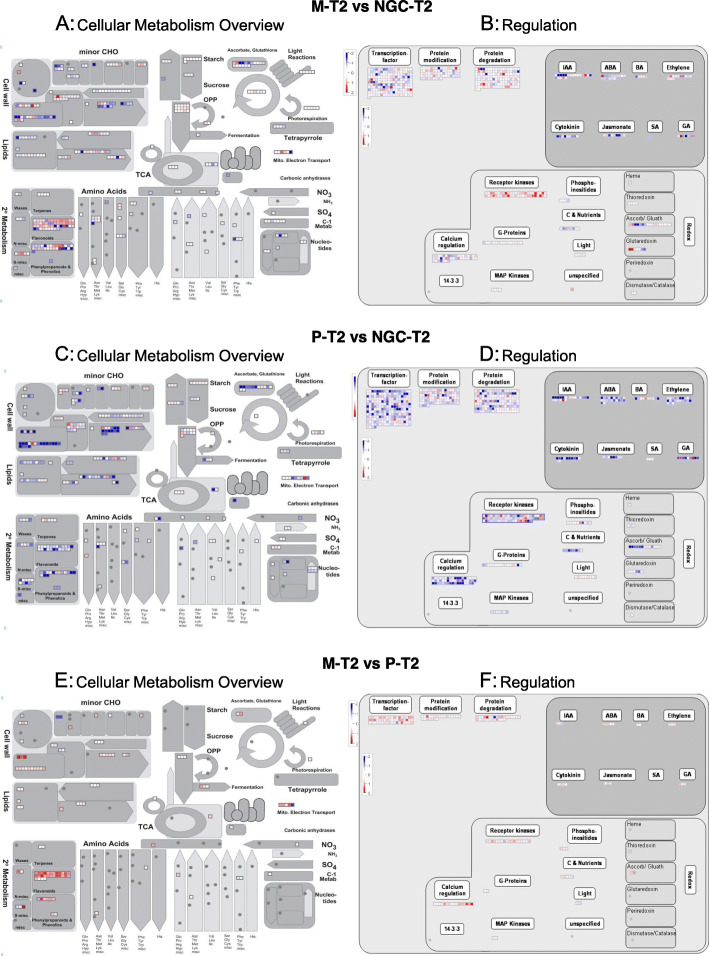
Panel **a**, **c**, **e**: Differences in the expression of genes involved in the cellular metabolism (metabolism overview) in the comparison at maturity (T2), visualized by MapMan. Each entity within a pathway is depicted by a color signal where red signifies genes with higher expression in the second sample compared to the first sample of the comparison (sample 1 vs sample 2), blue signifies genes with expression higher in the first sample of the comparison indicated on the graph. The intensity of the color indicates the level of expression. Scale bar displays log2 fold changes. Panel **b**, **d**, **f**: MapMan illustration depicting DEGs from the “Regulation” bins at maturity (T2). Log2 fold changes are indicated as a gradient of blue (up-regulated in the first sample, as indicated on the graph) and red (up-regulated in the second sample, as indicated on the graph) (PDF 452 kb)

MAPMAN analyses performed to evaluate metabolic pathways and cellular functions represented among differentially expressed genes confirmed the results obtained with GO analyses (Fig. 6). In particular, transcription factors and genes involved in protein degradation, modification, and signaling (receptor kinases and Ca^2+^ signalling) were modulated in T2 when comparing grafted and not grafted plants. Among transcription factors, the most represented families were MYB, bHLH, APETALA2/ERF, WRKY, Zinc-Finger, NAC, and some of them are well-known miRNA predicted targets. In detail, the P-T2 vs NGC-T2 comparison, showed the highest number of regulated TF, with 30 genes coding for MYB transcription factors and 20 WRKY domain transcription factors all but one up-regulated in P.

When comparing directly the two grafted plants at T2, most of the genes belonging to secondary metabolism, transcription factors, protein synthesis/degradation, and signaling were more expressed in plants grafted on 1103 Paulsen (P) than those grafted on Mgt 101–14 (M).

### Small RNA sequencing statistics and miRNA identification

We sequenced a total of 18 small RNA libraries, producing 124,548,127 raw redundant reads. After adapter trimming, we obtained 63,436,750 of which 50,892,703 ranging from 16 to 25 nt (Additional file 6).

Looking at the size distribution of the libraries (Additional file 7) we observed distinct peaks at 21 and 24 nt, as expected for DICER derived products. The 21 nt peak is the highest in all libraries indicating a preponderance of miRNA-like molecules while when considering the number of unique, non-redundant reads, the 24 nt peak is the highest showing a large variety of the siRNA-like molecules. It is worth noting that the 24 nt peak is much higher in berries at veraison (M-T1, P-T1, NGC-T1) than in mature berries (M-T2, P-T2, NGC-T2).

Clean and trimmed reads were used as input for miRNA identification and analyses, using CLC Bio Genomics Workbench software package. We performed a similarity search against miRNAs present in miRBase plus the user-defined dataset (see Methods). As a result, we identified 159 annotated MIR families. All the 48 grapevine MIR families have been retrieved. Additionally, 98 precursors of the 137 in the user-defined grapevine miRNAs have been retrieved in the sequencing data.

**Fig. 7:**
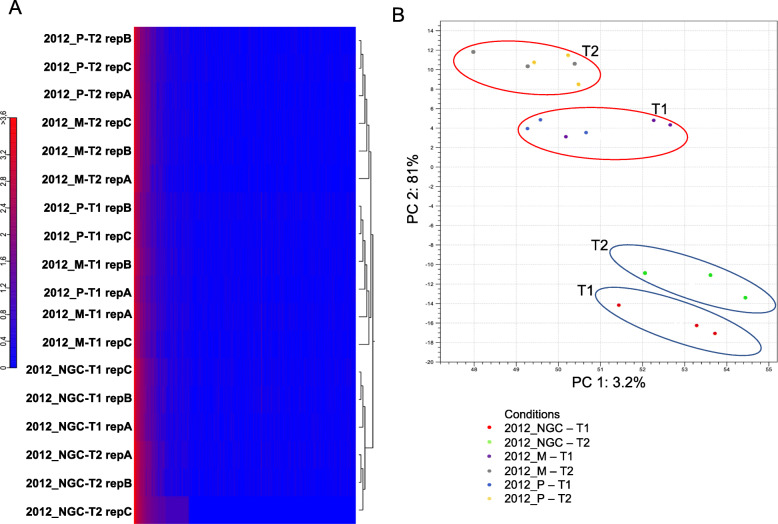
Panel **a:** Hierarchical cluster analysis (HCA) of all samples against small RNA-seq features. HC has been performed with normalized and log-transformed data, using 1-Pearson correlation as distance measure and Complete Linkage as linkage method. Colors in the heatmap, as indicated in the color key, represent miRNAs expression level, log-transformed. Panel **b**: Principal component analysis (PCA) of all 18 samples in the small RNA-seq dataset. The X-axis represents the first components and the Y-axis the second component. Each replicate of the same sample is associated with the same color. Blue ovals enclose NGC samples, red ovals enclose grafted samples (M and P). Sample names: M = Mgt 101–14; *P* = 1103 Paulsen; NGC = not grafted control; T1 = veraison; T2 = maturity (PDF 388 kb)

PCA and Hierarchical Clustering analysis (Fig. 7) were performed to monitor the quality of sample replicates and the overall similarity among samples: the analyses suggest a clear separation between grafted and not grafted vines and between T1 and T2.

### Differential expression and target identification of DE miRNAs

**Fig. 8:**
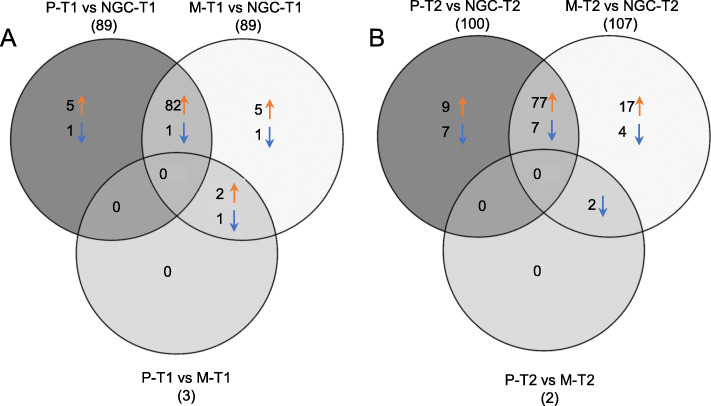
Venn diagram of differentially expressed miRNA sequences between the three root systems, at the same developmental stage (Panel **a**: T1 = veraison, Panel **b**: T2 = maturity). Total numbers of DE miRNAs are in brackets, number of up- and down-regulated miRNA features are indicated per each sub-set besides colored arrows. DEGs were called setting the FDR threshold at 0.05. Sample names: M = Mgt 101–14; *P* = 1103 Paulsen; NGC = not grafted control (PDF 91 kb)

Differential expression analysis of miRNA has been performed using CLC Bio software package, with all reads mapping to known plant miRNA precursors (miRBase Release 21 plus user-defined dataset). We focused our attention, as for transcriptomic analyses, to the comparisons among the three root systems, at the same developmental stage. The results of differential expression analyses (Fig. 8, and Additional file 8) indicate that the strongest differences arose when comparing grafted (either Mgt 101–14 or 1103 Paulsen) with not grafted control plants; most of the sequences were in common between the comparisons P-T1 vs NGC-T1 and M-T1 vs NGC-T1. Finally, almost all DE miRNAs were more expressed in not grafted plants than in grafted ones, at both veraison and maturity stages.

When comparing grafted plants directly (P-T1 vs M-T1 and P-T2 vs M-T2), only two or three sequences were differentially expressed at veraison and maturity, showing a minimal influence of different rootstocks on berry skin miRNAome.

On the whole, 98 and 123 sequences were differentially expressed at veraison and maturity, but it should be considered that more than one sequence may correspond to the same miRNA (isomiRNA), as indicated in Additional file 8. For each differentially expressed sequence, putative targets were identified in silico (Additional file 9).

Among the known miRNAs detected as differentially expressed between grafted and control plants, we found several miRNAs (e.g. miR482, miR535, miR396, miR3633, miR3632, miR3623, miR166, and miR159) regulating genes coding for proteins involved in disease resistance and TMV-resistance protein, putatively reinforcing the evidence coming from mRNA-seq data showing the class of abiotic/biotic stress response gene as differentially expressed at both T1 and T2. Apart from these, we focused our attention on those miRNAs with a putative function in secondary metabolism regulation, such as miR858, known to target MYB transcription factors regulating anthocyanins and flavonols, and two grapevine specific miRNAs [44, 46]: Grape_m-0721, targeting an anthocyanin 5-aromatic acyltransferase-like (VIT_213s0064g01165), and an anthocyanidin 5,3-O-glucosyltransferase (VIT_216s0050g00240), and Grape_m-1191 targeting an homologous to *TRANSPARENT TESTA 12* (*TT12* - VIT_212s0028g01160).

To enrich the analyses, for each DE miRNA, we cross-checked the expression profile of putative predicted targets in our transcriptomic data, confirming, for some of them, the opposite expression trend (Additional file 10), and reinforcing the role of those miRNAs as negative regulators of expression. For instance, miR156, in the comparison between 1103 Paulsen and not grafted plants at maturity, displayed an opposite expression profile compared to its predicted target VIT_211s0065g00170 (*VvSPL10 - Squamosa promoter-binding-like protein 12-like*) and one of the GRF targeted by miR396 (VIT_215s0048g01740) was up-regulated in Mgt 101–14 and 1103 Paulsen at maturity, showing an opposite expression trend compared to miR396. These miRNAs are well known to be implicated in grape berry development [44, 47].

Also for miR858, two sequence tags corresponding to ath-miR858a and ppe-miR858 were more expressed in not grafted plants than in grafted vines, both at T1 and T2 showing an opposite profile compared to three target *MYB* genes (*MYB174 -* VIT_218s0001g09850, *MYB175 -* VIT_218s0001g11170*,* and *MYB13* - VIT_205s0049g01010).

For some selected miRNAs, qRT-PCR was performed to validate the RNA-seq results (Additional file 11), but data were not confirmed, probably because of the presence of similar isomiR (one or two nt shorter) more expressed, and with a similar expression level among all the samples, that primers were not able to distinguish.

### Grape phenolic composition

Chemical analyses were carried out by HPLC to assess the concentrations of phenolic compounds in berry skins, as these molecules play a determinant role for wine quality. The results of the different metabolites detected are shown in Table 1. As expected, the general phenolic composition of berry skins was very different between veraison and maturity. The observed polyphenol abundance was in agreement with the common known trends of each metabolite class during grape ripening [26, 48].

**Table 1:** Phenolic compounds detected by HPLC in berry skin extracts. The values are expressed as HPLC areas. Different letters indicate significant differences (in bold) according to LSD multiple range test (95% confidence interval). Sample names: M = Mgt 101–14; *P* = 1103 Paulsen; NGC = not grafted control

	Veraison - T1			Maturity - T2			
	M	P	NGC	M	P	NGC	
Trans-caftaric acid	**868 a**	**894 a**	**1373 b**	584	547	623	**HYDROXYCINNAMIC ACIDS**
Cis-coutaric acid	**140 a**	**157 a**	**210 b**	64	66	80	
Trans-coutaric acid	**442 a**	**447 a**	**754 b**	296	265	303	
Epigallocatechin	**104 c**	**53 a**	**77 b**	22	45	42	**FLAVANOLS**
(+)-catechin	**59 b**	**23 a**	**39 ab**	17	48	19	
Myricetin-3-O-glucoside	**136 b**	**72 a**	**70 a**	207	208	145	**FLAVONOLS**
Quercetin-3-O-rutinoside	**59 a**	**40 a**	**101 b**	17	31	23	
Quercetin-3-O-galactoside	**91 b**	**34 a**	**95 b**	49	44	37	
Quercetin-3-O-glucuronide	**604 b**	**257 a**	**620 b**	306	280	251	
Quercetin-3-O-glucoside	**611 b**	**324 a**	**919 c**	**232 ab**	**255 b**	**159 a**	
Kaempferol-3-O-glucoside	**94 ab**	**46 a**	**119 b**	65	51	47	
Isorhamnetin-3-O-glucoside	**94 b**	**47 a**	**62 ab**	147	156	87	
Total anthocyanins	**7376 c**	**4853 b**	**1546 a**	**9047 b**	**11,230 b**	**5078 a**	**ANTHOCYANINS**
Disubstituted anthocyanins	**1711 c**	**1011 b**	**380 a**	**2530 b**	**3248 c**	**1430 a**	
Trisubstituted anthocyanins	**5666 c**	**3842 b**	**1166 a**	**6517 b**	**7982 b**	**3648 a**	
Trisubstituted/disubstituted anthocyanins ratio	3,74	3,41	3,33	2,59	2,47	2,54	
Delphinidin-3-glucoside	**474 c**	**258 b**	**90 a**	**341 b**	**408 b**	**168 a**	
Cyanidin-3-glucoside	**186 b**	**94 a**	**53 a**	**185 b**	**204 b**	**117 a**	
Petunidin-3-glucoside	**591 c**	**321 b**	**109 a**	**477 b**	**568 b**	**244 a**	
Peonidin-3-glucoside	**1525 c**	**916 b**	**327 a**	**2345 b**	**3044 c**	**1312 a**	
Malvidin-3-glucoside	**3263 b**	**4601 b**	**967 a**	**5698 b**	**7006 b**	**3236 a**	
Trans-piceid	42	28	39	**148 a**	**316 b**	**269 ab**	**STILBENES**
Trans-ε-viniferin	14	19	24	18	31	42	

At T1, higher diversity in the accumulation of several phenolic compounds (flavonols, flavanols, hydroxycinnamic acids, but also anthocyanins) was detected between M, P, and NGC samples. Since veraison is a transitory phenological phase, the differences here found may be due to minimal misalignments in berry ripeness degrees among the vines. At T2, significant differences emerged in the accumulation of anthocyanins and trans-piceid (a stilbene), whose synthesis increases considerably in skin tissues, approaching harvest time [26]. Being typically abundant molecules at maturity, the differences found between the three root systems were particularly interesting.

Based on our results, myricetin-3-O-glucoside, quercetin-3-O-glucoside, and quercetin-3-O-glucuronide were the most abundant flavonols in all the analyzed rootstock-scion combinations. Interestingly, both in P and M the amount of myricetin-3-O-glucoside raised at T2. In terms of anthocyanins, the concentration (both total anthocyanins and of every single anthocyanin) were significantly different among the root systems, both at T1 and at T2, with a greater similarity between M and P that show a high concentration of disubstituted anthocyanins (e.g. peonidin-3-O-glucoside) at T2. Considering stilbenes, the concentration of trans-piceid strongly increased and was detected as significantly different only at T2 in P vines.

**Fig. 9:**
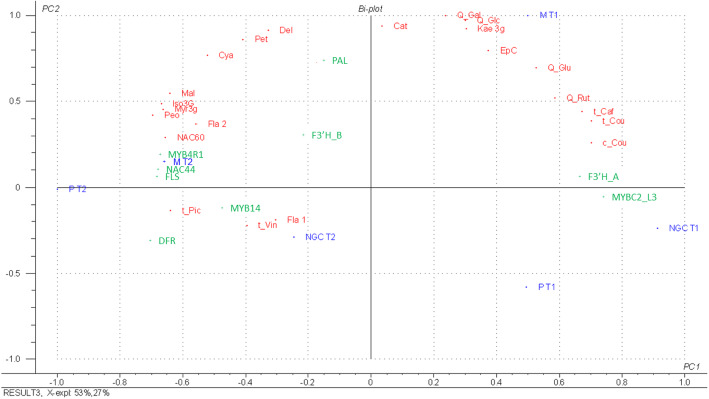
Principal Component Analysis (PCA) based on both qRT-PCR results and chemical analyses. Acronyms of phenolic compounds (in red): EpC = Epigallocatechin; Cat = (+)-catechin; t-Caf = trans-caftaric acid; c-Cou = cis-coutaric acid; t-Cou = trans-coutaric acid; Fla. 1 = unknown flavanol 1; Fla. 2 = unknown flavanol 2; Myr3g = myricetin-3-O-glucoside; Q-Rut = quercetin-3-O-rutinoside; Q-Gal = quercetin-3-O-galactoside; Q-Glc = quercetin-3-O-glucuronide; Q-Glu = quercetin-3-O-glucoside; Kae3g = kaempferol-3-O-glucoside; Iso3G = isorhamnetin-3-O-glucoside; Del = delphinin; Cya = cyanin; Pet = petunin; Peo = peonin; Mal = malvin; t-Pic = trans-piceid; t-Vin = trans-ε-viniferin. List of genes (in green): PAL (VIT_213s0019g04460), F3’H_A (VIT_209s0002g01090), F3’H_B (VIT_217s0000g07200), DFR (VIT_216s0039g02350), FLS (VIT_218s0001g03430), MYBC2-L3 (VIT_214s0006g01620), MYB14 (VIT_207s0005g03340), MYB4R1 (VIT_217s0000g02710), NAC44 (VIT_206s0004g00020), NAC60 (VIT_208s0007g07670). Sample names (in blue): M = Mgt 101–14; *P* = 1103 Paulsen; NGC = not grafted control; T1 = veraison; T2 = maturity (PDF 198 kb)

The PCA obtained considering both phenolic analyses and the expression of the genes involved in phenylpropanoid pathway (qRT-PCR data) confirmed some results already described, above all, the clear separation between the two maturation stages, with greater differentiation between the root systems at T1 (Fig. 9).

Further interesting results emerged considering the gene positions and the distribution of phenolic compounds in the PCA. At maturity, *MYB14*, *MYB4R1,* and *NAC44* genes are close to some stilbenes (*trans*-piceid and *trans*-ε-viniferin), confirming their key role in the biosynthesis of this class of compounds. The *PAL* and *F3’H* (VIT_217s0000g07200) genes, being in the central part of the graph, had a similar expression level both at T1 and T2, reflecting a constitutive activity during berry maturation.

## Discussion

### Standard growth conditions do not maximize the effect of different rootstocks at the molecular level

The present research was conceived to get information about the influence of a rootstock on grape quality and the results, overall, support an impact of rootstock on metabolite accumulation in the berries through modulation of gene expression. The experimental pot system employed was specifically designed to control most of the field variables (e.g. spatial variations across a field, irrigation, fertilization, pest control, environmental monitoring), with an adequate number of replicates, although simulating the real conditions of a vineyard. The choice of the cultivar *Pinot noir* and clone ENTAV115 was accurate, considering that the genome sequence of this cultivar/clone is completely annotated [1, 2], an important aspect, given the great varietal diversity within the *Vitis vinifera* species [49, 50]. Moreover, the use of pots made it possible to insert not grafted plants as a control, that would not be feasible in a real vineyard, due to the looming presence of phylloxera. According to Hierarchical Clustering and PCA on the transcriptomic data (Fig. 1 a-b), the pivotal effect leading gene expression was the berry developmental program, with a secondary effect of the rootstock, more evident at maturity. When comparing the samples at T2, the highest dissimilarity was detected between the grafted plants (P and M) and the not grafted control (NGC) with minor fluctuations in gene expressions comparing M and P. Considering miRNA expression, grafting is the main driving force that separated the samples, more than developmental plans (Fig. 7).

Grapevine physiology and berry ripening are highly influenced by temperature along the growing season [51] and evapotranspiration, which drives grapevine water status, increases in warmer climates enhancing the plants’ water demand [52]. Moderate stress conditions (like those recorded in 2012) can be beneficial, favoring an optimal maturation and stimulating the accumulation of secondary metabolites in red grape varieties without significantly compromising yield [26, 53, 54]. Despite the vines were maintained in optimal water conditions during the whole growing season, a clear influence of the rootstock on gene expression and metabolic responses was evident. These differences are much more significant in the case of water deficit because it is almost certain that the influence of the rootstock genotype on the physiological behavior of the scion becomes more appreciable in the presence of water stress [4, 10], strongly impacting on gene expression and phenolic compounds accumulation [55].

### Differentially expressed genes are mainly involved in secondary metabolism and its regulation

The differentially expressed genes and the enrichment analysis of their ontologies identified the major functional categories represented at T1 (M vs NGC and P vs NGC) as those related to photosynthesis, plastid organization, response to light stimulus (Fig. 5). Hence, at veraison, when berry skin color was shifting from green to purple, the photosynthetic activity decreased at a different rate and NGC showed a higher expression of photosynthetic genes, suggesting a residual photosynthetic activity. At T2, with a number of DEGs higher than in T1, the main biological processes differentially regulated were hormonal changes, the response to biotic or abiotic stresses, and secondary metabolism, phenylpropanoid pathway, and cell wall biosynthesis, all processes connected to berry ripening and softening (Fig. 5 and Fig. 6). In general, the genes related to these classes were more expressed in P than in NGC or M; when comparing NGC and M plants, the genes of secondary metabolism class were mostly up-regulated in NGC, while genes of other ontologies were predominantly up-regulated in M (Fig. 6). Taken together, these data show that berries grown on the 1103 Paulsen rootstock had a higher expression, at T2, of genes involved in secondary metabolism suggesting a stronger influence of 1103 Paulsen rootstock than Mgt 101–14 or not grafted control plants on ripening processes related to secondary metabolite accumulations in berries.

In addition, many genes coding for transcription factors (mainly belonging to MYB, bHLH, and WRKY families, the key regulators of the phenylpropanoid pathway [56, 57]) were clearly differentially regulated (Fig. 6).

Forty-one genes belonging to MYB/bHLH families were mainly up-regulated in P-T2 compared to NGC-T2, reinforcing the hypothesis of a strong modulation effect coming from this rootstock genotype on the berry phenolic contents in the scion.

We further analyzed the results coming from small RNA sequencing data to highlight their putative involvement in secondary metabolism. In this perspective, it is interesting to show the results of vvi-miR858, a miRNA already identified in *Arabidopsis*, apple, and peach [58–60]. Although not deposited in miRBase for *Vitis vinifera*, it has already been reported in previous works [42, 61] and it has been validated via degradome analysis as one of the master regulators of *MYB* genes in grapevine berries [62]. Indeed, we predicted among its targets, 34 R2R3-MYB transcription factors (Additional file 9) including three *MYB* genes (*MYB174 -* VIT_218s0001g09850, *MYB175 -* VIT_218s0001g11170, and *MYB13 -* VIT_205s0049g01010) identified as DE in the comparison M vs NGC and P vs NGC, with an opposite expression profile compared to miR858 (Additional file 10), reinforcing the idea that MYB transcription factors regulating secondary metabolism might be modulated by rootstock effect.

At maturity, in particular in the comparison P vs NGC, another miRNA, miR156, showed an opposite expression profile compared to its SPL target. miR156 is well known to be up-regulated during grape ripening, while its target SPL decreases [44, 47, 63]. While in grapevine SPL genes have not yet been functionally characterized, in *Arabidopsis* and tomato they are known to target MADS-box genes involved in fruit development [64, 65] and, moreover, AtSPL9 has been demonstrated to negatively regulate anthocyanin biosynthesis [66]. This evidence, together with our data, would suggest that 1103 Paulsen has a less severe SPL down-regulation in berries at maturity, compared to NGC plants, hence maintaining its activity on downstream pathways.

Unfortunately, the results of qRT-PCR (Additional file 11) did not coincide with those of RNA-seq, because it was impossible to distinguish among the DE and not DE isomiR of the miR858. An interesting fact, however, is that at veraison miR858 was more expressed than at maturity suggesting that the translation of the mRNAs coding for the MYBs involved in the secondary metabolism was most likely inhibited at T1 and favored at T2.

### Biochemical and molecular data support the rootstock effect on phenolic compounds accumulation in berry skins

The accumulation of some phenolic compounds in berry skins (Table 1 and Fig. 9), confirmed some major trends already described. Berry skins differed for flavonoids accumulation between T1 and T2 [26, 30] and the data highlighted minor but significative differences between the two grafted vines.

The PCA run merging metabolic and qRT-PCR data showed a correlation between gene expression and concentrations of phenolic compounds, confirming the role of some genes in the biosynthesis of specific flavonoid molecules. *VvDFR* (VIT_216s0039g02350), a DIHYDROFLAVONOL-4-REDUCTASE, is the enzyme that carries out the first step of anthocyanidins synthesis, converting dihydroflavonols into leucoanthocyanidins. The substrate of DFR is common with FLS (flavonol synthase), and between the two enzymes, there is a dichotomy for the alternative synthesis of anthocyanins and proanthocyanidins or flavonols [67]. *DFR* showed higher expression in P and M at T2 (Fig. 4), and even though it’s only a trend not statistically significant it may suggest that grafted plants had a greater aptitude for anthocyanins accumulation in berry skins towards maturity. This result is confirmed by the higher amount of anthocyanins in M and P vines at T2 (Table 1).

Besides structural genes, different classes of transcription factors were differentially regulated among P, M and NGC plants. We found MYB transcription factors, which finely control the phenylpropanoid synthesis pathway in grapevine [68] and includes several members, both positive or negative regulators, most of which have been largely characterized in grapevine.

The *VvMYB14* gene (VIT_07s0005g03340) is involved in the feedback regulation of resveratrol biosynthesis, a branch of the phenylpropanoid pathway that leads to stilbene accumulation [69]. It is known that abiotic stresses induce *VvMYB14* that up-regulates the activity of the *VvSTS29* gene (stilbene synthase), resulting in resveratrol accumulation. On the contrary, when resveratrol level increases, *VvMYB14* is down-regulated, preventing the accumulation of this metabolite [70]. The gene coding for the MYB14 transcription factor was up-regulated in 1103 Paulsen at T2, and the expression was almost doubled compared to M and NGC plants (Fig. 4). Furthermore, according to HPLC data, P vines accumulated a higher concentration of trans-piceid at T2, the major resveratrol derivative in grapes [71], and the close placement of MYB14 and trans-piceid in the PCA that merged qRT-PCR and grape phenolic composition data (Fig. 9) supported this hypothesis. These results suggest that the plants grafted on 1103 Paulsen have a greater predisposition to the synthesis of MYB14, which could induce a greater accumulation of resveratrol in mature berries. This hypothesis could explain the effect of a greater tolerance to drought given by this commonly used rootstock. In a work published by Corso et al. [22], the transcript profiles of two rootstocks with opposite drought susceptibility were compared (Mgt 101–14, the same as the present work and M4, a new drought-tolerant rootstock); according to their findings, MYB genes (including *MYB14*) were found as DE between the rootstock genotypes under water stress, both in leaves and roots. The MYB family was one of the most represented among the DE genes and had opposite expression kinetics between Mgt 101–14 and the drought-resistant rootstock M4, which has intrinsic characteristics very similar to 1103 Paulsen in stress tolerance.

The *MYBC2-L3* (VIT_214s0006g01620) is a transcriptional repressor in the anthocyanin synthesis [72]. In transgenic tobacco [73], *VvMYBC2-L3* represses the *DFR* gene and might induce the expression of *FLS*, although this latter hypothesis was not confirmed. *MYBC2-L3* was not DE in the current experiment, neither at T1 nor at T2, but is listed among the predicted targets of vvi-miR858 (Additional file 9). The expression of this repressor was higher at veraison than at maturity. We can hypothesize that, at T1, the up-regulation of *MYBC2-L3* promoted the flavonols synthesis to the detriment of anthocyanins in grape berries, while at T2 the lower expression of *MYBC2-L3* repressor favored the accumulation of anthocyanins. This result is supported by HPLC data: at T1 the concentrations of most flavonols were higher and decayed at T2, whereas the concentration of anthocyanins considerably grew from T1 to T2.

Among the different *NAC* genes described in grapevine [74], *VvNAC44* (VIT_206s0004g00020) and *VvNAC 60* (VIT_208s0007g07670) were the most interesting genes to be counted among the DEGs. They seem to be involved in berry ripening and stress response [75–77], and according to our results, both from RNAseq and qRT-PCR, *VvNAC44* and *VvNAC60* were less expressed in NGC vines than in grafted ones at T2 (Fig. 4) suggesting that grafting on 1103 Paulsen and Mgt 101–14 had an influence on these transcription factors and, more in general, in berry ripening processes.

## Conclusions

Although grafting has an essential role in viticulture, the molecular network behind the rootstock-scion interaction remains largely unknown, particularly concerning grape quality. Our data confirmed that, even without a severe stress that may exacerbate the differences, rootstocks can determine important effects on grape phenotype, affecting the final berry quality. We also observed that grafting per se has an influence on berry skin transcriptome and chemical composition at maturity. The genes identified as differentially expressed at maturity were mainly involved in the synthesis of phenylpropanoids and in the transport of flavonoids. Besides, the secondary metabolism was more significantly modulated during grape ripening in the plants grafted on 1103 Paulsen than in those grafted on Mgt 101–14. The vines grafted on 1103 Paulsen had a greater predisposition to the synthesis of MYB14 compared to Mgt 101–14, which could induce a greater accumulation of resveratrol and its derivatives in mature berries, as observed with HPLC data on trans-piceid.

In the light of the results obtained, we can conclude that rootstocks may influence the molecular mechanisms of berry development and grape quality.

## Methods

### Plant materials

A pot system for grapevines monitoring was set up at CREA - Research Centre for Viticulture and Enology, in Arezzo (43°28′36″ N, 11°49′27″ E, Italy). It consists of plastic pots of 70 l, filled by a silty-clay texture soil (40% clay, 41% silt, 19% sand), with a volumetric soil water content of 34% at field capacity, collected from a real vineyard of the Chianti Classico D.O.C.G. district (Tuscany - Italy). The grapevines in the pots were 6-year-old *Pinot noir* plants, clone ENTAV 115 with two different rootstock combinations: 1103 Paulsen (P) *V. berlandieri* x *V. rupestris*, highly vigorous and known for its drought tolerance, and Mgt 101–14 (M) *V. riparia* x *V. rupestris*, less vigorous and less tolerant to drought; not grafted plants were used as control (NGC). The vines were trained on vertical shoot positioned trellis, with spur cordon pruning and an average of 10 buds per vine. The pots were positioned in an open field, spaced at the distance of 1 m within the row and 2.5 m between the rows, with orientation north to south, and were arranged in a randomized block design with 9 replicates for each root system. The vines were maintained in the same agronomic conditions: all the pots were fertilized before the beginning of the vegetative season with 40 g of Nitrophoska (12 N-12P-17 K, EurochemAgro) and were abundantly irrigated by drip emitters during the summer period, with the same water regime.

In 2012, grape samples for molecular analyses were collected at two ripening times: veraison (75% of colored berries, T1) and at maturity (maturity, T2). Berries (15 per plant, 3 plants per replicate) were randomly hand-picked at different positions of the clusters, dissected to separate skin tissues, stored in Falcon tubes and immediately frozen at − 80 °C for further processing. In total, the experiment entailed the collection of 18 different samples (vines with three different root systems, two ripening times, and three biological replicates), each comprising the skins of 15 berries.

At harvest, technological maturity was evaluated on musts according to O.I.V. official methods [78], confirming commercial ripeness; no significant differences emerged between the grape samples (average data: sugars 22.2 °Brix, pH 3.6, total acidity 6.7 g/L tartaric acid, berry weight 0.9 g).

### Plant specimen

The plant material used belongs to *Vitis vinifera* species or hybrid species of *Vitis* commonly used in viticulture and freely available for cultivation or research activity. In particular, *Pinot noir* is officially registered in the Italian National Catalogue of Grape Varieties (identification code n°195 - admitted in 1970). The information is available at the following link: http://catalogoviti.politicheagricole.it/result.php?codice=195. *Pinot noir* clone ENTAV 115 is officially registered in the French National Catalogue of Grape Varieties (admitted in 1971). The information is available at the following link: http://plantgrape.plantnet-project.org/it/cepage/Pinot%20noir. The rootstock 1103 Paulsen is officially registered in the Italian National Catalogue of Grape Varieties (identification code n°625 - admitted in 1971). The information is available at the following link: http://catalogoviti.politicheagricole.it/result.php?codice=625. The rootstock Mgt 101–14 is officially registered in the Italian National Catalogue of Grape Varieties (identification code n°604 - admitted in 1971). The information is available at the following link: http://catalogoviti.politicheagricole.it/result.php?codice=604.

The plant materials employed in the experimental pot system was preventively genotyped using a set of nine SSR loci internationally recognized for grapevine identification (http://www.oiv.int/public/medias/6886/oiv-viti-609-2019-en.pdf). Furthermore, the identity of the *Pinot noir* cultivar was confirmed by repeated ampelographic surveys on shoots, leaves, bunches, and berries.

### Weather conditions

The climate data were recorded during the 2012 vegetative season using a non-stop automated control unit (Ecotech GmbH, Germany) placed nearby the experimental pot system area. The following parameters were measured in the period comprised between April 1st and October 31st (conventionally considered the vegetative period for the grapevine): daily maximum temperature (°C), daily average temperature (°C), daily minimum temperature (°C); daily rainfall (mm). The data collected were daily checked and processed for each year at the end of the season; Growing degree days (GDDs) and the Winkler Index were calculated on a 10 °C based temperature, according to Winkler [79], to get information about the sum of all the daily average temperatures that influenced the plant growth during the season. The data recorded are reported in Additional file 1.

### Library preparation and sequencing

Total RNA extraction from the berry skins of 18 samples (three root systems per two ripening times per three biological replicates) was performed using Plant RNA Isolation Reagent (PRIR – Life Technologies™) starting from 200 mg of ground tissue in 1 mL of reagent, followed by RNA Clean up and Concentration kit (NorgenBiotek Corp) according to manufacturers’ protocols. Total RNA was then subjected to Dnase I treatment (DNA-free™ Kit, Applied Biosystems). The concentration and purity of total RNAs were evaluated using a spectrophotometer (DU640 Beckman) and a Nanodrop 2000 Spectrophotometer (Thermo Scientific) and their integrity was assessed by an Agilent 2100 Bioanalyzer using an RNA 6000 Nano kit (Agilent Technologies), according to the manufacturer’s instructions. All RNA samples were stored at − 80 °C for subsequent analyses.

Small RNA libraries were prepared using the TruSeq Small RNA Sample Preparation Kit (Illumina®), following all manufacturers’ instructions. Eighteen bar-coded small RNA libraries were constructed starting from 1 μg of total RNAs. The quality of each library was assessed using an Agilent DNA 1000 kit. Sequencing was performed using a 6-plex sequencing approach on an Illumina GAIIx platform.

mRNA seq libraries were prepared from the same total RNA (1 μg) extracted for small RNA ones, using TruSeq RNA sample preparation kit (Illumina®), according to manufacturers’ instructions. Libraries were quantified through qRT-PCR, as recommended by the protocol, and single-end sequenced for 100 bases on an Illumina Genome Analyzer (GAIIx).

### Bioinformatics and statistical methods

#### miRNAs methods

Identification and quantification of grapevine miRNAs have been carried out with the software CLC Bio Genomics Workbench (v.8, Qiagen). Using this software, raw redundant reads have been processed to trim the adapter. Reads between 16 and 25 nt were retained and compared (zero mismatches) with all plant species miRNAs deposited in miRBase v.21 (www.mirbase.org) [80, 81], and, additionally, with a set of 139 novel grapevine miRNAs (user-defined dataset) identified in our previous works [44, 46]. Differentially expressed miRNAs were identified using the software CLC Bio Genomics Workbench using the Empirical analysis of DGE tool and the multiple comparison analysis. For each library, each ungrouped read perfectly mapping to the miRNA precursors was considered as the input for the expression analysis. We then considered for further analyses DE reads overlapping (+/− 5 nt) the 5′ and 3′ mature miRNA only.

Given the main focus of our work, we aimed at identifying miRNAs differentially expressed between the two grafted plants and among grafted and control plants, sampled at the same developmental stage. We performed the Empirical Analysis of digital gene expression (DGE), an implementation of the “Exact Test” present in the EdgeR Bioconductor package [82], as implemented in CLC Bio Genomics Workbench software. We estimated tagwise dispersion with a multi-comparison unpaired test option, setting the FDR-adjusted *p*-value < 0.05. We classified the differentially expressed sequences based on the miRNA family they belong to, and on the correspondence to the mature 5′ or 3′ miRNA product or the position into the precursor stem-loop structure.

PCA and Hierarchical Clustering analyses have been performed, using all the reads mapping on the precursors, within the software CLC Bio Genomics Workbench, using normalized (tag per 1 million TP1M) and transformed data (log_10_ (n + 1)), where n is the normalized value for each sequencing tag. Hierarchical Clustering analyses have been performed using 1-Pearson correlation as distance measure and Complete Linkage as the linkage method.

All differentially expressed sequences have been used as input for psRNATarget software (https://plantgrn.noble.org/psRNATarget/analysis) [83], to predict putative target sequences for each DE miRNA, from Grapevine transcript database originated from JGI - Phytozome v11 (https://phytozome.jgi.doe.gov/pz/portal.html) [84] and Genoscope 12x assembly [1]. Default settings have been used to run analyses, modifying HSP size for sequences shorter than 20 nt.

#### RNA-Seq, differentially expressed genes, GO enrichment and further methods

Raw reads (101 bases, single end; on average 21 million of reads for each sample, Additional file 2) were checked for adapters and contaminants via FastQC application [85]. Adapters and low-quality regions were filtered out by Cutadapt application [86]. Subsequently, TopHat version 2.0.12 and Bowtie2 [87, 88] were implemented to map filtered reads to the grapevine genome sequence (*Vitis vinifera* [1]; Vitis_vinifera.IGGP_12x.25). Read counts were generated from Bam alignment files with HTSeq software version 0.6.1 [89]. Data normalization and call of differentially expressed genes (DEGs) was implemented with DESeq2 version 1.2.8 Bioconductor (R) package [90] by setting fitting to local, and False Discovery Rate (FDR, Benjamini-Hochberg multiple test correction) threshold to 0.05 and enabling independent filtering. No fold change threshold was set.

GO enrichment analyses were conducted with the Goseq Bioconductor package. Goseq was specifically designed to minimize length-derived bias which may affect RNA-seq data [91]. Data preparation for Goseq analysis was as previously reported [92].

MapMan [93] figures were generated upon binning of *Vitis* cDNA sequences to MapMan bins by the Mercator application [94]. PCA of samples were based on R function prcomp from stats package as implemented in DESeq2 Bioconductor package.

### qRT-PCR analyses of miRNAs and gene expression

miRNAs expression levels were evaluated by stem-loop Real-Time PCR (qRT-PCR); the primers (listed in Table 2) were designed according to Varkonyi-Gasic [95]. For reverse transcription, a stem-loop primer for each miRNA was used. Stem-loop reverse transcriptase primers consist of a selfed stem-loop sequence, in addition to a specific nucleotide extension at the 3′ end, complementary to the last 6 nucleotides at the 3′ end of each miRNA of interest.

**Table 2:** List of forward, reverse and stem-loop reverse transcriptase primers used for qRT-PCR to test genes and miRNAs expression. Gene ID/miRNA sequences are specified

Gene/miRNA	Gene ID/miRNA sequence	Forward Primer (5′3’ Seq.)	Reverse Primer (5′3’ Seq.)	Stem-loop reverse transcriptase Primer (5′3’ Seq.)
*VvPAL*	VIT_213s0019g04460	CGCCAAACACAGCCACTCA	GCAGCTTTAGTACCAGTGTCTCCC	–
*VvF3’H*	VIT_209s0002g01090	TCCTACCACCTCACCAACGC	CGAGAGGAGGATAAGAGCCACAGT	–
*VvF3’H*	VIT_217s0000g07200	GCCTCCGTTGCTGCTCAGTT	CGTAGGGAGCGAACACCAGA	–
*VvFLS*	VIT_218s0001g03430	TTGATATCCCACGACACACCG	ATTGAGATCAGCACCAGAGGC	–
*VvDFR*	VIT_216s0039g02350	TGAGAAGGAGAAACATGCATGCCA	AGGTGACCCATTGCAACTTTCA	–
*VvMYB14*	VIT_207s0005g03340	CGGAGAGCCTTGGGTATGGA	TGCAGGGTGTAGTAATGTCGGA	–
*VvMYBC2-L3*	VIT_214s0006g01620	CTCACCATTGCCATTCCTGCT	AGGATTTGCGTCACCTTCCAC	–
*VvMYB4R1*	VIT_217s0000g02710	CCTCTCTCATTGAAGCCGCTC	GTTTCTGGATTGCACGGAGGA	–
*VvNAC44*	VIT_206s0004g00020	GGACGACTGGGTTCTTTGCC	CCATCGTCTTCAGCCACCTC	–
*VvNAC60*	VIT_208s0007g07670	ACGTTCGAGCATGGATGGG	CTTTGCGGGAGGTCTGACTG	–
*VvUBI*	VIT_219s0177g00040	AATGGTCAGTTGGCCCTACCT	TGGCTGAGACCCACAAAACC	–
Vvi-miR395	CTGAAGTGTTTGGGGGAACTC	TGACGCTGAAGTGTTTGGGG	GTGCAGGGAGGGAGGT	GTCGTATCCAGTGCAGGGAGGGAGGTATTCGCACTGGATACGACGAGTTC
Vvi-miR398	TGTGTTCTCAGGTCGCCCCTG	TCGCTTGTGTTCTCAGGTCG	GTGCAGGGAGGGAGGT	GTCGTATCCAGTGCAGGGAGGGAGGTATTCGCACTGGATACGACCAGGGG
Grape_m-0721	TTACCAACACCTCCCATTCC	TGCGGATTACCAACACCTCC	GAGCTGGGTCCGACGT	GTCGTATCCAGAGCTGGGTCCGACGTATTCGCTCTGGATACGACGGAATG
Grape_m-1191	GCTGAACAAGAGAGAACCT	GCGCGGCTGAACAAGAGA	GAGCTGGGTCCGACGT	GTCGTATCCAGAGCTGGGTCCGACGTATTCGCTCTGGATACGACAGGTTC
Vvi-miR858	CGTTGTCTGTTCGACCTTG	TCGCCCGTTGTCTGTTCG	GTGCAGGGTCCGAGGT	GTCGTATCCAGTGCAGGGTCCGAGGTATTCGCACTGGATACGACCAAGGT

The RT reactions were performed starting from 200 ng of DNase treated total RNA, using Superscript III (Invitrogen), according to the manufacturer’s instructions. The reverse transcription products were amplified using a miRNA-specific forward primer and a reverse primer on the stem-loop adapter.

The Real-Time PCR reactions were set up in 25 μL using SYBR Green PCR Master Mix (Applied Biosystem). Three independent biological replicates were analyzed in triplicate, on a 7300 Real-Time PCR System (Life Technologies™) with the following conditions: 95 °C for 10 min, followed by 40 cycles of 95 °C for 15 s and 60 °C for 1 min plus 1 cycle for dissociation curve. A poly-ubiquitin transcript (*VvUBI* - VIT_219s0177g00040) was always used as an internal standard [21]. After the amplification, the 7300 Sequence Detection System Software was used to set the baseline and the threshold for each reaction. The relative quantification of each miRNA was calculated from the Ct value, using the 2^-ΔΔCt^ method [96].

To evaluate gene expression level, primers were designed in non-conserved coding regions (Table 2) to avoid cross-amplification of genes belonging to multigenic families; primer efficiency was calculated using serial dilutions of berry skin cDNA. cDNA was produced from DNase-treated RNA using SuperScript II Reverse transcriptase kit (Invitrogen) according to manufacturer’s instructions.

Before the setting up of qRT-PCR on the chosen genes, the efficiency of the 10 pairs of primers, previously designed (Table 2) was tested with successful results on serial dilutions of berry skin cDNA. The Real-Time PCRs were performed in a final volume of 10 μL, with SsoAdvanced Universal SYBR® Green PCR Supermix (BioRad), considering three technical replicates for each sample. The plates were analyzed on a 7300 Real-Time PCR System (Life Technologies) with the following conditions: 95 °C for 30 s, followed by 40 cycles of 95 °C for 10 s and 60 °C for 1 min plus 1 cycle for primer dissociation. After the amplification, the 7300 Sequence Detection System Software was used to set the baseline and the threshold for each reaction. The relative quantification was calculated from average Ct value, using the 2^-ΔΔCt^ method [96], considering a poly-ubiquitin transcript (*VvUBI* - VIT_219s0177g00040) as an internal standard [21].

### Chemical analyses

The phenotyping activity was carried out on grape quality, in particular on the content of phenolic compounds in berry skins. The samples (15 berries per plant, 3 plants per replicate) were collected simultaneously for molecular and chemical analyses at veraison (T1) and maturity (T2). The skin tissues were separated and immediately ground into a powder using a mortar and liquid nitrogen, then were stored at − 80 °C in falcon tubes, until use.

Before analyzing, the berry skin powder was weighed and resuspended in 10 mL of methanol (ultra) gradient HPLC grade (JT Baker, USA) and 50 μL of formic acid 98% (PanreacApplichem, Spain). The solution was centrifuged at 3000 rpm for 10 min and then 2 mL of extract were pipetted into a syringe, filtered with Minisart RC 0.45 μm filters (Sartorius, Germany), and injected into HPLC glass vials. The analyses were performed using an Agilent 1100 Series HPLC, equipped with solvent degasser, quaternary pump and diode array detector and controlled by a PC running Agilent ChemStation for LC 3D System software (Agilent, USA). A Luna® Omega 5 μm Polar C18 Column (Phenomenex, USA) was used to separate phenolic compounds, following the method of Gomez-Alonso [97]. In total, the experiment comprised 18 berry samples (vines with three different root systems, two ripening times, three biological replicates). The results obtained as HPLC output were processed using the software Statgraphics (Statgraphics Technologies Inc., USA). In particular, the data were subjected to one-way analysis of variance (ANOVA); statistically significant differences were assumed for *P* < 0.05. The mean values were then separated by the LSD multiple range test (95% confidence interval). To merge HPLC and qRT-PCR data, a Principal Component Analyses was obtained using the software Unscrambler (V10.3, CAMO Process AS, Norway).

## Supplementary information

**Additional file 1.** Weather conditions (April 1st - October 31st) . Rainfall = daily rainfall (mm); T max = daily maximum temperature (°C); T avg. = daily average temperature (°C); T min = daily minimum temperature (°C); DGGs = Growing degree days; DOY = day of the year. The grey arrow indicates the veraison sampling date (T1); the black arrow indicates the maturity sampling date (T2).

**Additional file 2.** Raw reads and mapping statistics for RNA-seq libraries.

**Additional file 3.** Correlation coefficient among replicates and samples.

**Additional file 4.** List of differentially expressed genes, indicating for each gene in each comparison FDR, Log2 Fold Change, expression level for each sample as the output of DESeq2 and Blast2GO field description (Folder containing 6 TSV files 4.47 Mb).

**Additional file 5 **Venn diagrams of enriched GO terms (Biological Processes) in the three comparison considered at veraison - T1 (Panel A) and maturity - T2 (Panel B). Sample names: M = Mgt 101–14; *P* = 1103 Paulsen; NGC = not grafted control.

**Additional file 6.** Raw reads and trimming statistics for small RNA-seq libraries.

**Additional file 7.** Size distribution of sequencing reads, between 16 and 25 nt, for each sample sequenced by small RNA seq. For each sample, it is reported the number of unique-different sequences, and the total number (redundant) of sequences of a given length.

**Additional file 8.** List of differentially expressed sequence tag, for small RNA seq. For each sequence is given: length, reference miRNA and the reference species, the miRNA type (5′ or 3′, exact match or shifted) average normalized abundance, log2 Fold Change and FDR.

**Additional file 9.** Target predicted in silico (psRNA Target), for each differentially expressed sequence in small RNA seq data.

**Additional file 10.** List of differentially expressed miRNAs (as calculated by small RNAseq analysis) and their relative differentially expressed targets (as calculated by RNAseq data analysis). For each miRNA/target pair is reported the comparison considered, target id putative function and GO terms, miRNA name and sequence, and log2 fold change of the target and the miRNA. Only statistically significant DEG and DE miRNAs are reported.

**Additional file 11 **Expression profiles of the 5 selected miRNAs obtained by qRT-PCR, calculation from Ct value with the 2^-ΔΔCt^ method (the bars indicate the standard error). Sample names: M = Mgt 101–14; *P* = 1103 Paulsen; NGC = not grafted control; T1 = veraison; T2 = maturity.

## Data Availability

The datasets supporting the results of this publication are included in the article and its Additional files. All the sequencing data have been submitted to the Archive ArrayExpress (EMBL-EBI), with the following ID accession numbers: E-MTAB-8758 for RNAseq data, and E-MTAB-8756 for small RNA data.

## Abbreviations

ath: *Arabidopsis thaliana*
bHLH: Basic helix-loop-helix
Ct: Threshold cycle
DE: Differentially expressed
DEG: Differentially expressed gene
DFR: Dihydroflavonol-4-Reductase
D.O.C.G.: Denomination of controlled and guaranteed origin
DOY: Day of the year
ERF: Ethylene response factor
F3’H: Flavonoid 3′-hydroxylase
F3’5’H: Flavonoid 3′,5′-hydroxylase
FDR: False discovery rate
FLS: Flavonol synthase
GDD: Growing degree days
GO: Gene ontology
GRF: Growth regulating factor
HC/HCA: Hierarchical clustering analysis
HPLC: High-performance liquid chromatography
L.: *Linnaeus*
M: Mgt 101–14
MIR genes: Genes coding for microRNA
miRNA/miR: microRNA
NAC: NAM-ATAF1,2-CUC2
NGC: Not grafted control
nt: Nucleotide
O.I.V.: International organization of vine and wine
P: 1103 Paulsen
PAL: Phenylalanine ammonia lyase
PCA: Principal component analysis
ppe: *Prunus persica*
PTGS: Post transcriptional gene silencing
qRT-PCR: Quantitative real time PCR
rpm: row per minute
siRNA: Short interfering RNA
SPL: Squamosa promoter-binding-like
SSR: Simple sequence repeats
STS: Stilbene syntase
T1: Time 1, veraison
T2: Time 2, maturity
TF: Transcription Factor
TT: Transparent Testa
UBI: Ubiquitin
UV: Ultraviolet
vs: Versus
Vv/vvi: *Vitis vinifera*
