# Supplementary figures and images for: Transcriptomic and biochemical investigations support the role of rootstock-scion interaction in grapevine berry quality
Source: BMC Genomics. 2020 Jul 8;21:468. doi: 10.1186/s12864-020-06795-5 (PMC7341580; doi:10.1186/s12864-020-06795-5)

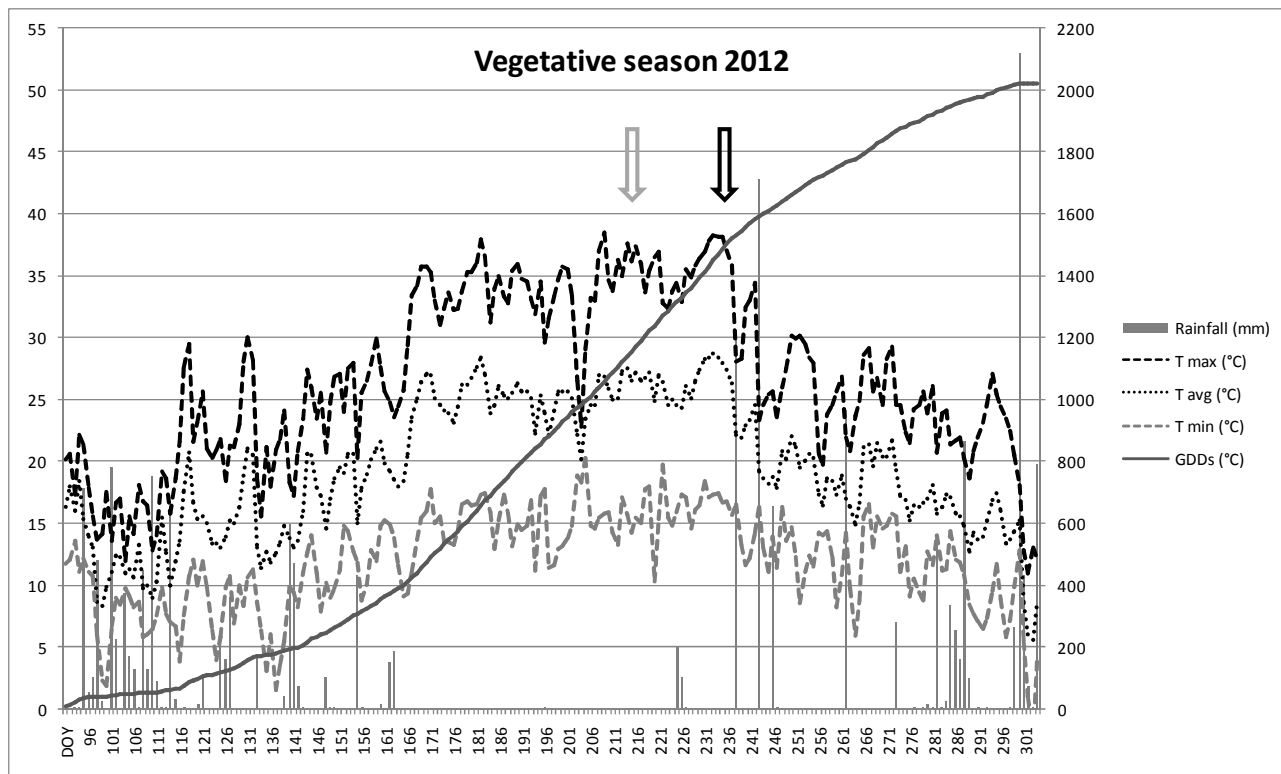

Supplement: Supplementary file 1 — Additional file 1. Weather conditions (April 1st - October 31st) . Rainfall = daily rainfall (mm); T max = daily maximum temperature (°C); T avg. = daily average temperature (°C); T min = daily minimum temperature (°C); DGGs = Growing degree days; DOY = day of the year. The grey arrow indicates the veraison sampling date (T1); the black arrow indicates the maturity sampling date (T2). [file 12864_2020_6795_MOESM1_ESM.pdf]

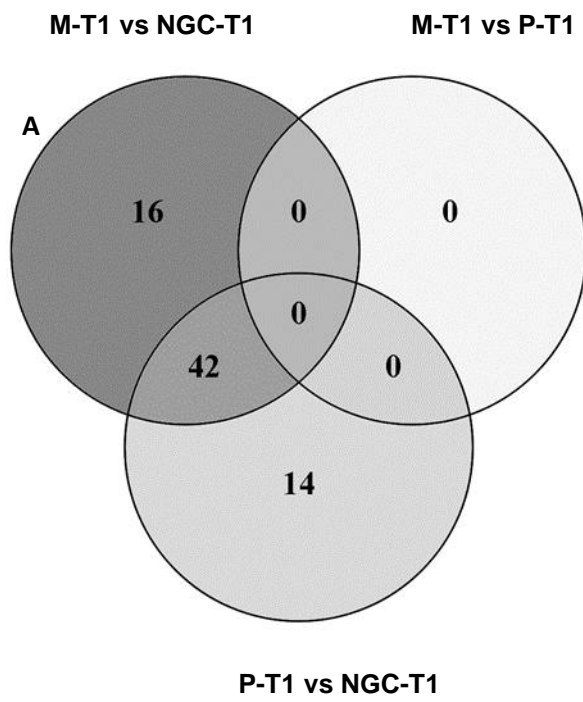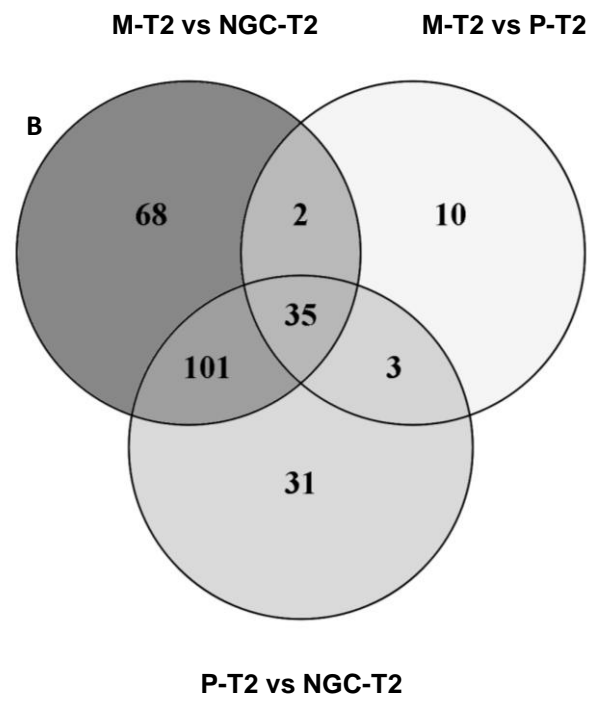

Supplement: Supplementary file 5 — Additional file 5 Venn diagrams of enriched GO terms (Biological Processes) in the three comparison considered at veraison - T1 (Panel A) and maturity - T2 (Panel B). Sample names: M = Mgt 101–14; P = 1103 Paulsen; NGC = not grafted control. [file 12864_2020_6795_MOESM5_ESM.pdf]

Size distribution- 2012 season- unique reads

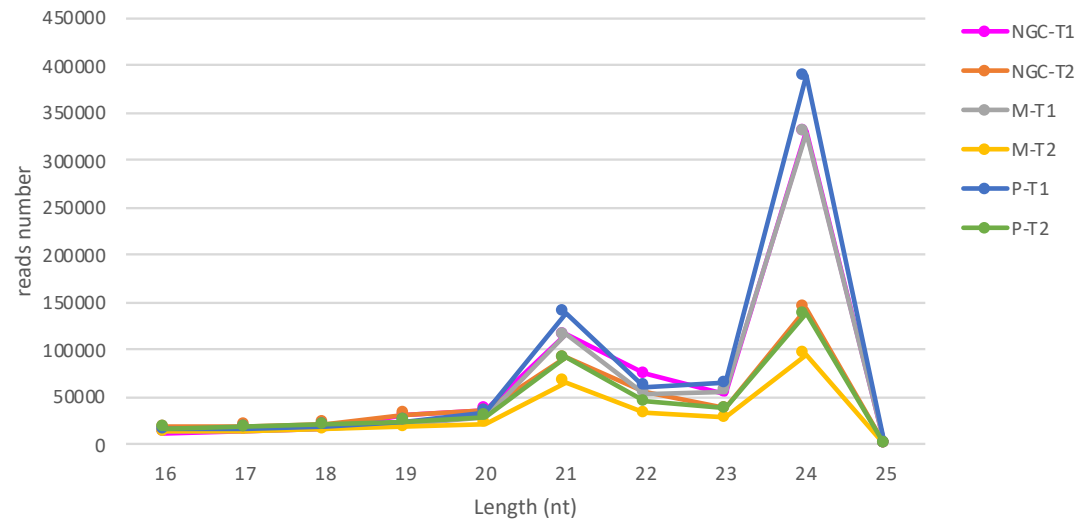

size distribution -2012- redundant

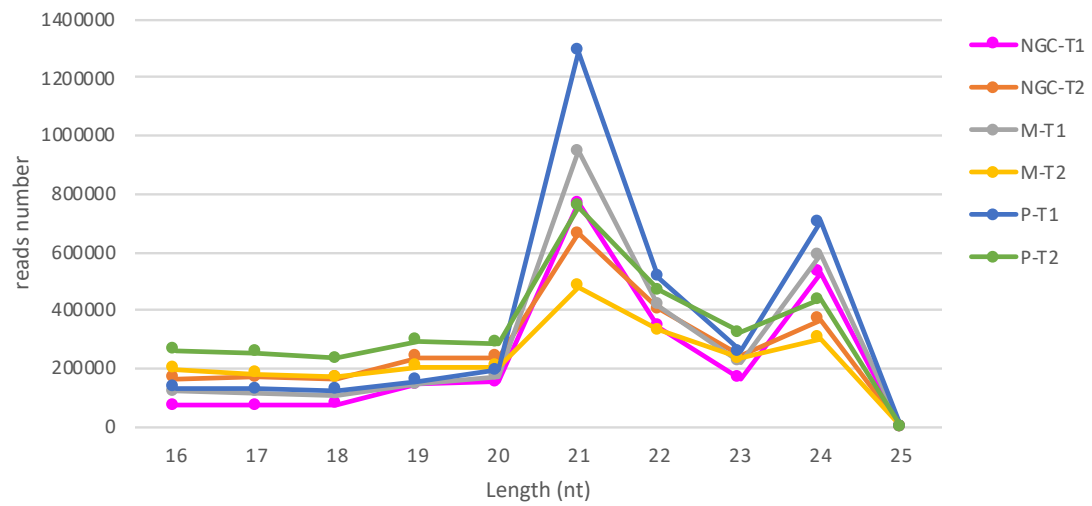

Supplement: Supplementary file 7 — Additional file 7. Size distribution of sequencing reads, between 16 and 25 nt, for each sample sequenced by small RNA seq. For each sample, it is reported the number of unique-different sequences, and the total number (redundant) of sequences of a given length. [file 12864_2020_6795_MOESM7_ESM.pdf]

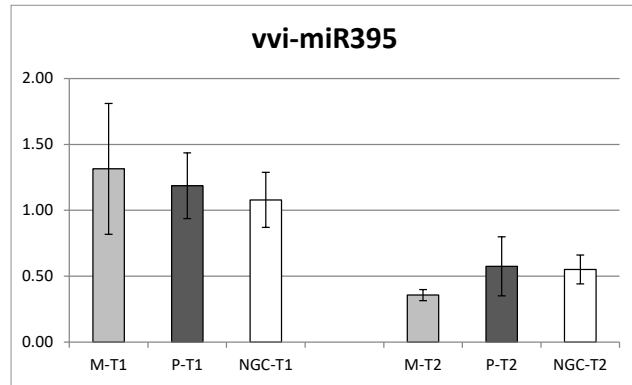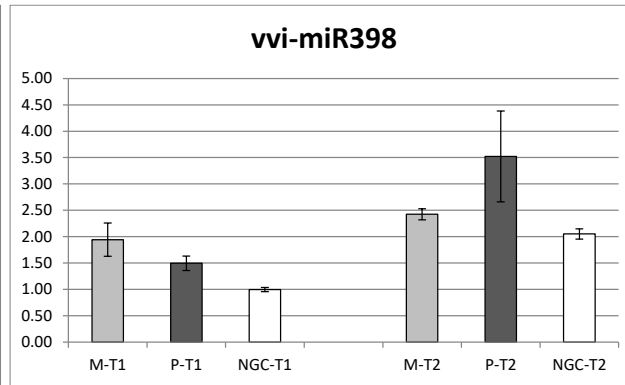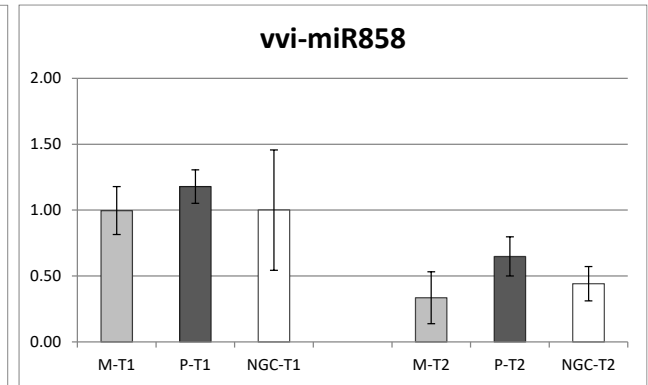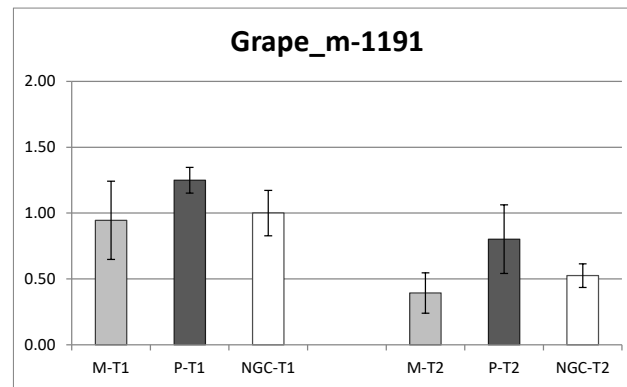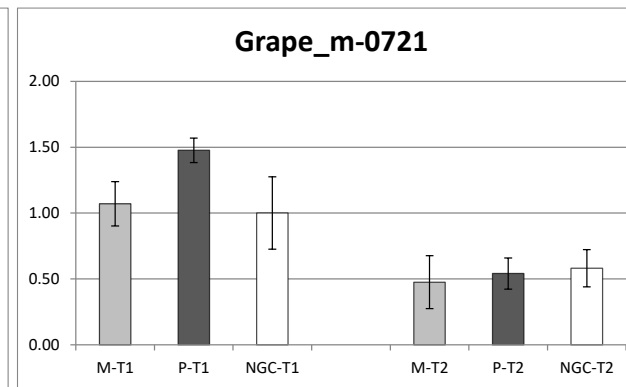

Supplement: Supplementary file 11 — Additional file 11 Expression profiles of the 5 selected miRNAs obtained by qRT-PCR, calculation from Ct value with the 2-ΔΔCt method (the bars indicate the standard error). Sample names: M = Mgt 101–14; P = 1103 Paulsen; NGC = not grafted control; T1 = veraison; T2 = maturity. [file 12864_2020_6795_MOESM11_ESM.pdf]
